# Supplementary material for: Long-range exciton transport and slow annihilation in two-dimensional hybrid perovskites
Source: Nat Commun. 2020 Jan 31;11:664. doi: 10.1038/s41467-020-14403-z (PMC6994693; doi:10.1038/s41467-020-14403-z)
Supplement: Supplementary file 1 — Supplementary Information [file 41467_2020_14403_MOESM1_ESM.pdf]

# Long-Range Exciton Transport and Slow Annihilation in Two-Dimensional Hybrid Perovskites

Deng *et al*.

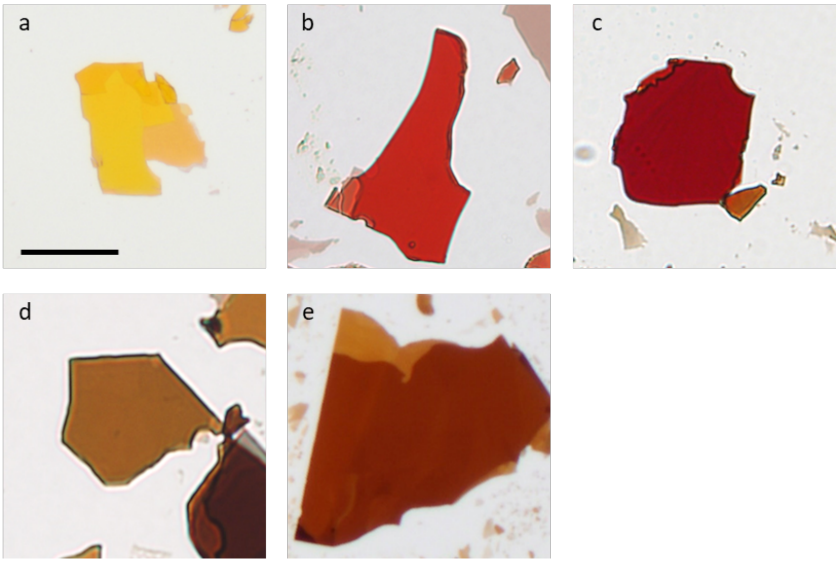


**Supplementary Figure 1** | Transmitted optical images of $(BA)_2(MA)_{n-1}Pb_nI_{3n+1}$ on fused silica with $n$ = 1 (**a**), $n$ = 2 (**b**), $n$ = 3 (**c**), $n$ = 4 (**d**), $n$ = 5 (**e**). The scale bar is 20 μm.

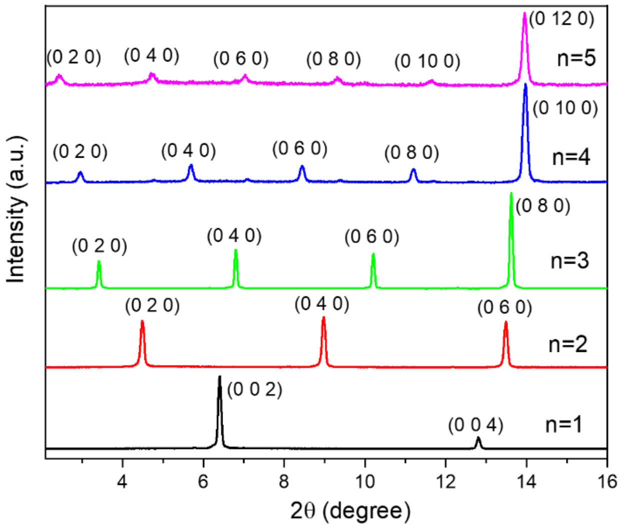


**Supplementary Figure 2 |** XRD profiles of exfoliated $(BA)_2(MA)_{n-1}Pb_nI_{3n+1}$. The inorganic layers are shown to be parallel to the substrate surfaces.

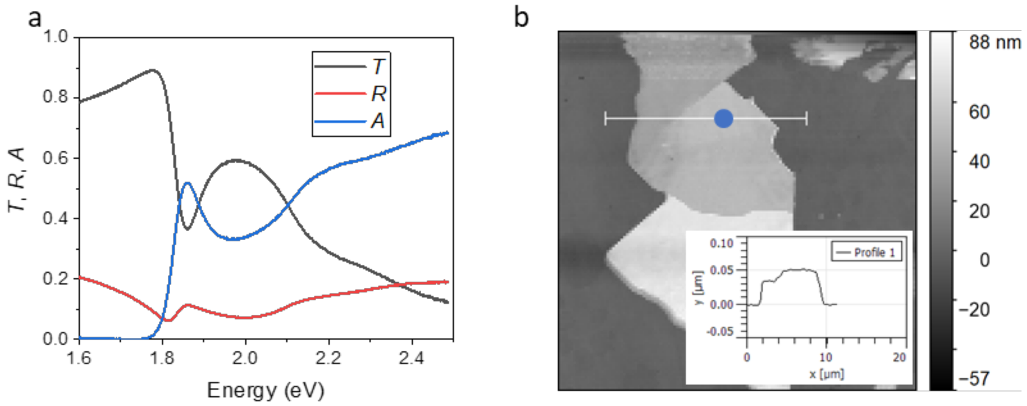


**Supplementary Figure 3** | Reflective and transmitted spectra (**a**) and AFM image (**b**) of $(BA)_2(MA)_4Pb_5I_{16}$ flakes. The spectra were collected at the blue spot. Image size is 20 × 20 μm.

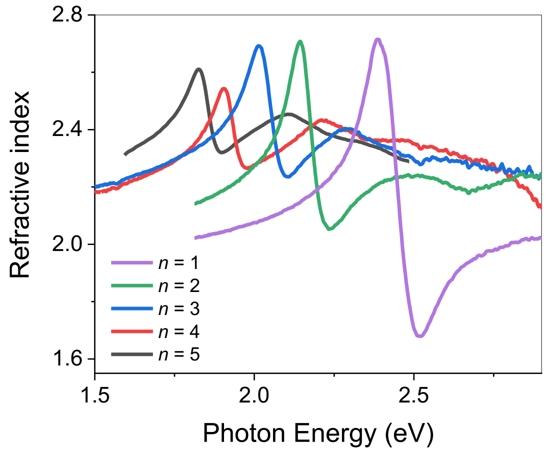


**Supplementary Figure 4 |** Refractive index of $(BA)_2(MA)_{n-1}Pb_nI_{3n+1}$ with *n* varying from 1 to 5.

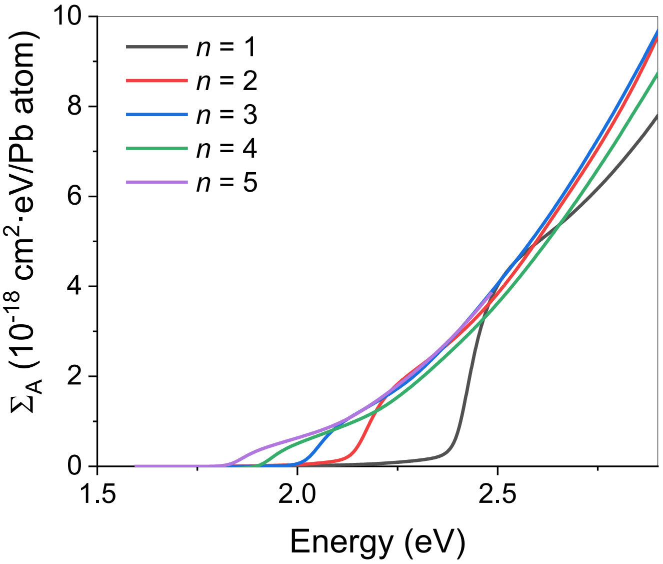


**Supplementary Figure 5 |** Integrated absorption cross section per Pb atom of $(BA)_2(MA)_{n-1}Pb_nI_{3n+1}$ with $n$ varying from 1 to 5.

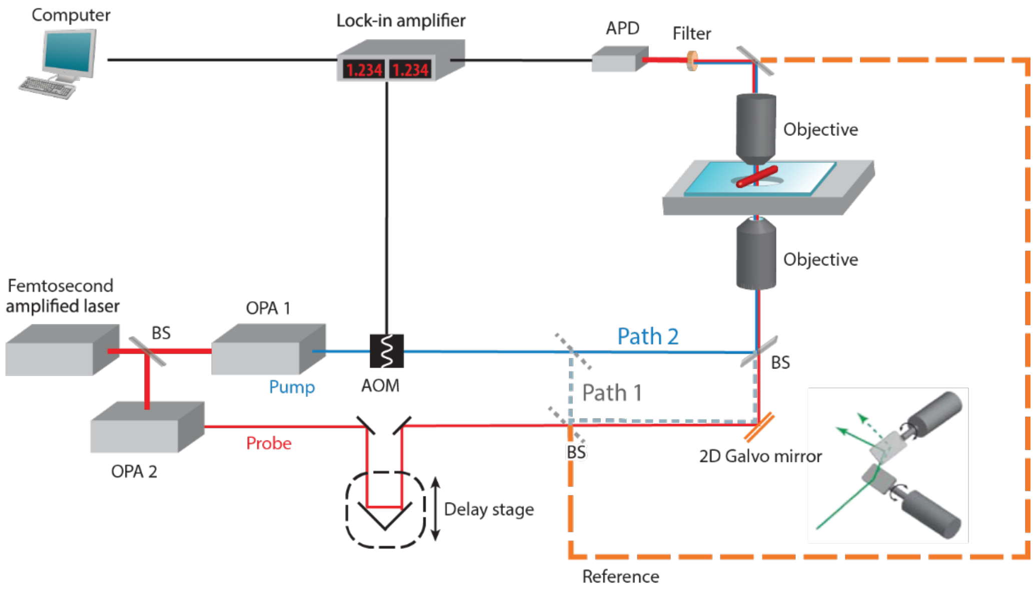


**Supplementary Figure 6 |** Schematic representation of a TAM setup. In path 1 (dashed gray line) the pump and probe beams are scanned together in space and the spatially dependent dynamics are imaged. In path 2 (blue line) only the probe beam is scanned to construct images of the excited state population. Abbreviations: AOM, acoustic-optical modulator; APD, avalanche photodiode; BS, beam splitter; OPA, optical parametric amplifier; TAM, transient absorption microscopy.

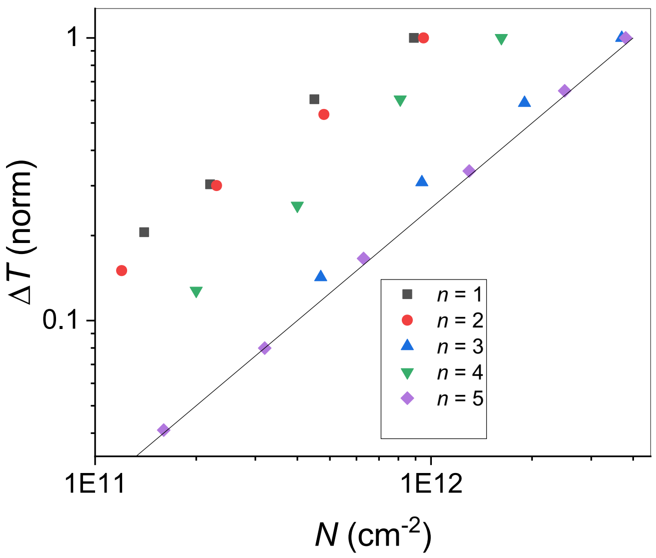


**Supplementary Figure 7 |** Transient absorption signal intensity at ground state bleach as a function of carrier density of $(BA)_2(MA)_{n-1}Pb_nI_{3n+1}$ with *n* varying from 1 to 5.

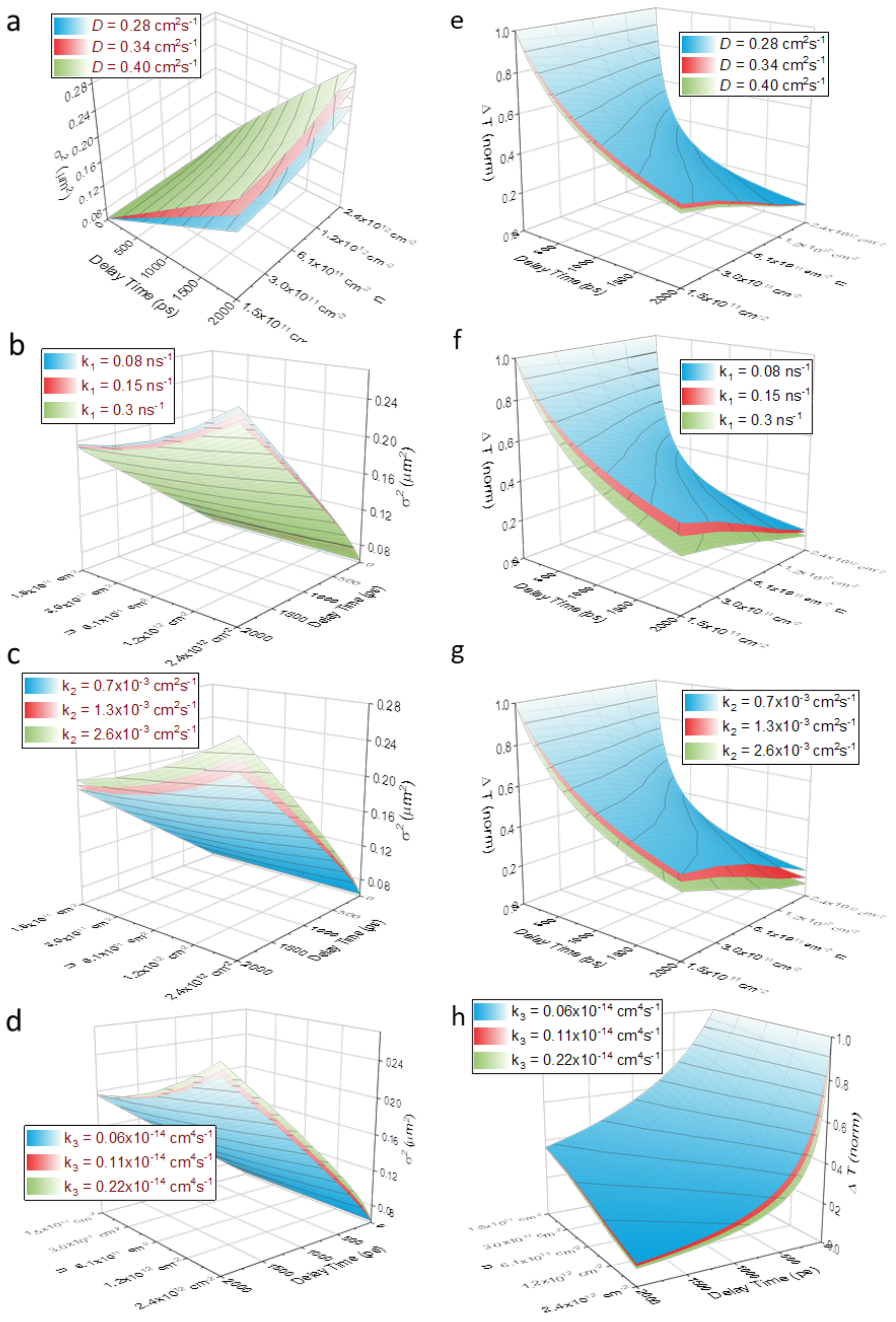


**Supplementary Figure 8 |** Sensitivity analysis of the model to each parameter, showing the $\sigma^2(D, k_1, k_2, k_3)$ and $\Delta T(D, k_1, k_2, k_3)$ for different values of $D$ (**a**, **e**), $k_1$ (**b**, **f**), $k_2$ (**c**, **g**) and $k_3$ (**d**, **h**) around the median parameters. The variation of each parameter acts differently on $\sigma^2(D, k_1, k_2, k_3)$ and $\Delta T(D, k_1, k_2, k_3)$ ensuring the independence of the determined parameters.

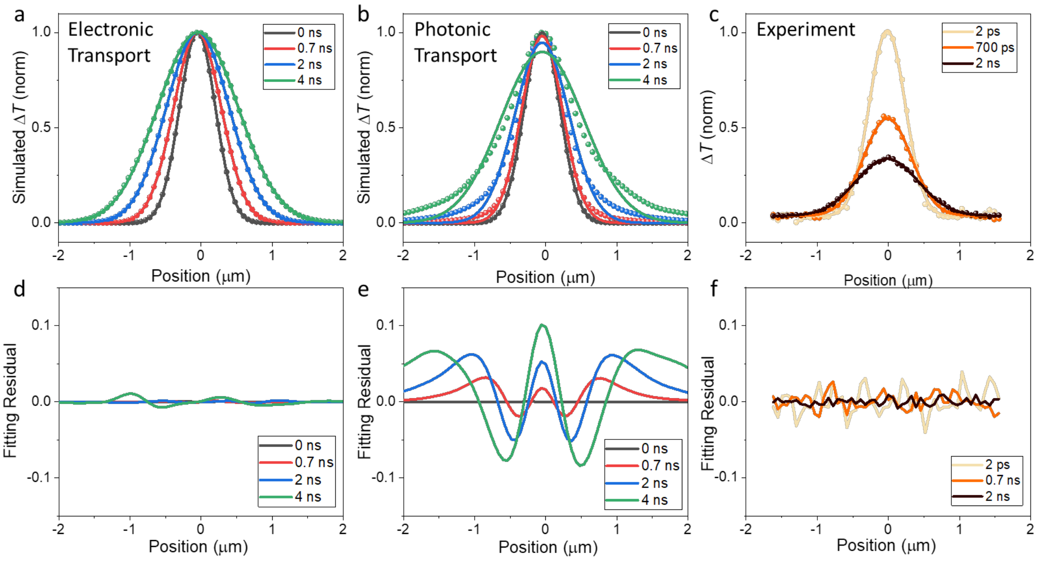


**Supplementary Figure 9** | Monte Carlo simulation of carrier transport. **a-c** simulated TAM profile for electronic transport (**a**), photonic transport (**b**) and experimental TAM profile (**c**), the curves in (**a**) and (**b**) are normalized to its maximum, the curves in (**c**) are normalized to the maximum of the curve at 2 ps. **d-f** the residual of Gaussian fitting for (**a-c**).

| parameter | value | parameter | value |
| --- | --- | --- | --- |
| $T_{\mathrm{c}}$ | 300 K | $\alpha$ | 0.2×10$^5$ cm$^{-1}$ |
| $m_{\mathrm{exciton}}$ | $m_e$ | $\tau_{\mathrm{p}}$ | 1 ns |
| $\tau_{\mathrm{s}}$ | 7.5 fs | $\sigma_{\mathrm{pump}}$ | 0.15 µm |
| | | $\sigma_{\mathrm{probe}}$ | 0.2 µm |

**Supplementary Table 1**. Parameters used in the simulation shown in Supplementary Figure 9.

**Supplementary Note 1. Determination of the linear extinction coefficient $\alpha$ and the refractive index**

For a thin film on thick transparent substrate, the reflectance and transmittance of normal incident light are given by the Fresnel's equations [1]

$$R = \frac{(n_0^2+\boldsymbol{n}_1^2)(\boldsymbol{n}_1^2+n_2^2)-4n_0\boldsymbol{n}_1^2n_2+(n_0^2-\boldsymbol{n}_1^2)(\boldsymbol{n}_1^2-n_2^2)\cos 2\delta_1}{(n_0^2+\boldsymbol{n}_1^2)(\boldsymbol{n}_1^2+n_2^2)+4n_0\boldsymbol{n}_1^2n_2+(n_0^2-\boldsymbol{n}_1^2)(\boldsymbol{n}_1^2-n_2^2)\cos 2\delta_1} \quad (1)$$

$$T = \frac{8n_0\boldsymbol{n}_1^2n_2}{(n_0^2+\boldsymbol{n}_1^2)(\boldsymbol{n}_1^2+n_2^2)+4n_0\boldsymbol{n}_1^2n_2+(n_0^2-\boldsymbol{n}_1^2)(\boldsymbol{n}_1^2-n_2^2)\cos 2\delta_1} \quad (2)$$

Where $\delta_1 = \frac{2\pi}{\lambda}\boldsymbol{n}_1 d_1$, $n_0$, $\boldsymbol{n}_1$ and $n_2$ are the refractive index of air, the thin film and the substrate, respectively. $d_1$ is the thickness of the thin film, $\lambda$ is the wavelength. Once the $R$ and $T$ are measured for a specific $\lambda$ and $d_1$, one can determine the real part ($n$) and imaginary part ($\kappa$) of $\boldsymbol{n}_1$ by resolving Supplementary Equation 1 and 2. Then linear extinction coefficient $\alpha$ can be obtained by $\alpha = \frac{4\pi\kappa}{\lambda}$.

**Supplementary Note 2. The uncertainty of TAM measurements**

There are two main sources of noise contributing to the TAM imaging: laser fluctuation noise and electronic noise from the detection system (for example, detector and lock-in amplifier). Noise due to laser intensity fluctuations can be effectively eliminated by using heterodyne lock-in detection with MHz modulation where the intensity of the excitation beam (or additional local oscillator) is modulated by an acoustic-optical modulator. Subsequently, a lock-in amplifier referenced to this modulation frequency can sensitively extract the induced signal. The fluctuation of laser intensity ($1/f$ noise) usually occurs at low frequency (lower than 10 kHz). In our experiments, we use a modulation frequency of 100 kHz.

For the exciton transport measurements, we use TAM to track the movement of excitons in real space, and the diffusion coefficient is determined using the equation

$$2Dt = L^2 = \sigma_t^2 - \sigma_0^2$$

One may perform sensitivity analysis by differentiating the above expression and find that

$$\Delta L=\sqrt{\frac{\sigma_t^2}{\sigma_t^2-\sigma_0^2}\left(\Delta\sigma_t\right)^2+\frac{\sigma_0^2}{\sigma_t^2-\sigma_0^2}\left(\Delta\sigma_0\right)^2}=\sqrt{\Delta\sigma_t^2+\left(\frac{\sigma_0}{L}\right)^2\left(\Delta\sigma_0^2-\Delta\sigma_t^2\right)}$$

This shows that the error comes from the uncertainty of the width for Gaussian profiles measured at $t$, which is determined by the signal-to-noise of the TAM measurements. A similar discussion on the uncertainty of such imaging approach can be found in a prior work by Akselrod *et al.*[2], where a photoluminescence microscopy imaging technique was employed.

**Supplementary Note 3. Differentiate the electronic and photonic contributions to exciton diffusion in 2D perovskite**

Both electronic mechanism and photonic mechanism can contribute to the carrier transport[3, 4]. The TAM technique we use in this paper not only helps us to determine the diffusion coefficient but also offers a chance to differentiate the electronic and photonic contribution by examining the spatial distribution of carriers.

Microscopically, the electronic mechanism and photonic mechanism can be described by two slightly different random walk pictures which will be detailed later in next paragraph. For the electronic mechanism, each step size of the random walk is determined by the electronic mean free path which is in the order of nm. To achieve a final average transport distance of 100s of nm, every carrier needs to walk $10^5$ steps randomly, this will result in a Gaussian type distribution of transport distance. For the photonic mechanism, the step size is determined by the penetration depth $\alpha^{-1}$ which is in the order of 100s of nm. To achieve a final average transport distance of 100s of nm, the hoping steps of carriers will be quite discrete like 0, 1 and 2 which will result in a distribution of transport distance strongly deviates from Gaussian type. Here we perform Monte Carlo simulations to visualize this difference.

**Electronic mechanism.** Exciton gas with a temperature $T_{\mathrm{c}}$ is created with the initial positions of carriers randomized by Gaussian distribution with standard deviation $\sigma_{\mathrm{pump}}$. The excitons undergo a random walk in the crystal due to the scattering process.

The travel direction $\theta$ is randomized for each step, the speed is determined by the kinetic energy, the flight duration between two scattering is randomized with the time constant of $\tau_{\mathrm{s}}$.

**Photonic mechanism.** Excitons are created with the initial positions randomized by Gaussian distribution with standard deviation of $\sigma_{\mathrm{pump}}$. The excitons undergo a random hoping in the crystal by emission-reabsorption process. The hoping direction $\theta$ is randomized for each step, the step size is randomized with the penetration depth $\alpha^{-1}$, the waiting time between two hoping is randomized with the time constant of $\tau_{\mathrm{p}}$.

In both simulations, the spatial distributions at specific times are recorded for $10^4$ excitons, convoluted with probe profile (Gaussian type with standard deviation $\sigma_{\mathrm{probe}}$) to simulate TAM imaging. The result is shown in Supplementary Fig. 8, the parameters used are listed in Supplementary Table 1.

For electronic mechanism, the spatial distribution of excitons at each delay time is Gaussian type which can be determined by the unstructured residuals of Gaussian fittings. For photonic mechanism, the spatial distributions of excitons at each delay time strongly deviate from Gaussian; thus, the residuals of Gaussian fittings are highly structured. We replot the curves in Fig. 2b and in Supplementary Fig. 8c for comparison, their fitting residuals (Supplementary Fig. 8f) are unstructured, which means the spatial distribution of excitons is Gaussian type. Therefore, we conclude that photonic contributions from photon recycling in exciton transport is negligible.

The photonic contribution can be highly affected by (1) PLQY; (2) geometry of the sample; (3) dielectric mismatch between sample and environment. Under the right conditions, we expect exciton transport can be enhanced using photonic mechanism over the intrinsic electronic transport we measured.

## Supplementary Note 4. Synthesis of 2D halide perovskite crystals and sample preparation [5-7]

### 4.1 $(BA)_2PbI_4$ ($n$ = 1) crystals

The solid precursors, including PbO (0.57 mmol) and BAI (0.57 mmol), were dissolved

into a mixture acid containing 0.9 mL of HI and 0.1 mL of $H_3PO_2$ in a 10 mL glass vial. With magnet stirring, this vial was heated to 120 $^oC$ in oil bath. After the solid precursors were completely dissolved and the solution becomes transparent, the stirring was terminated, and the solution was cooled down at a rate of 10 $^oC$ per 5 min. The crystals nucleated and grew during the cooling process. Finally, the crystals were collected by vacuum infiltration and the residue solvent was removed via vacuum pumping.

4.2 $(BA)_2MAPb_2I_7$ ($n$ = 2) crystals

The solid precursors, including PbO (0.59 mmol), BAI (0.43 mmol) and MAI (0.31 mmol), were dissolved into a mixture acid containing 0.9 mL of HI and 0.1 mL of $H_3PO_2$ in a 10 mL glass vial. Other procedures were similar to $n$ = 1.

4.3 $(BA)_2(MA)_2Pb_3I_{10}$ ($n$ = 3) crystals

The solid precursors, including PbO (0.59 mmol), BAI (0.19 mmol) and MAI (0.40 mmol), were dissolved into a mixture acid containing 0.9 mL of HI and 0.1 mL of $H_3PO_2$ in a 10 mL glass vial. Other procedures were similar to $n$ = 1.

4.4 $(BA)_2(MA)_3Pb_4I_{13}$ ($n$ = 4) crystals

The solid precursors, including PbO (0.69 mmol), BAI (0.17 mmol) and MAI (0.52 mmol), were dissolved into a mixture acid containing 0.9 mL of HI and 0.1 mL of $H_3PO_2$ in a 10 mL glass vial. Other procedures were similar to $n$ = 1.

4.5 $(BA)_2(MA)_4Pb_5I_{16}$ ($n$ = 5) crystals

The solid precursors, including PbO (0.59 mmol), BAI (0.118 mmol) and MAI (0.472 mmol), were dissolved into a mixture acid containing 0.89 mL of HI and 0.1 mL of $H_3PO_2$ in a 10 mL glass vial. Other procedures were similar to $n$ = 1.

**Mechanical exfoliation of 2D halide perovskites**

Commercial Scotch tapes (3M) were used to exfoliate 2D halide perovskite crystals. Firstly, the solution-synthesized perovskite crystals were sandwiched between the adhesive sides of two Scotch tape. Then, these two tapes were separated to exfoliate the crystal. This process was repeated for 5 to 10 times. Finally, either of these two tapes

was pressed on a fused silica substrate. After the tape was removed, perovskite thin crystals were left on the substrate. The mechanical exfoliation was performed in $N_2$-filled glovebox. The exfoliated halide perovskite crystals are parallel to the $[PbI_6]^{4-}$ octahedral layers, as illustrated from the XRD profiles.

**Epoxy encapsulation of exfoliated perovskite thin crystals**

Firstly, the substrate containing exfoliated perovskite thin crystals was placed on a 0.17 mm-thick square coverslip, with the crystal side facing the coverslip. Then, the epoxy glue was added to the edges of the substrate to seal the crystals between the substrate and the coverslip. The drying of the epoxy generally takes about 1 h. This encapsulation process was performed in $N_2$ filled glovebox.

## Supplementary References:


1. Heavens, O. S. *Optical Properties of Thin Solid Films*, 1st edn. Dover Publications (1991).

2. Akselrod, G. M.*, et al.* Visualization of exciton transport in ordered and disordered molecular solids. *Nat. Commun.* **5**, 3646 (2014).

3. Gan, Z.*, et al.* The dominant energy transport pathway in halide perovskites: photon recycling or carrier diffusion? *Adv. Energy Mater.* **9**, 1900185 (2019).

4. Bercegol, A.*, et al.* Quantitative optical assessment of photonic and electronic properties in halide perovskite. *Nat. Commun.* **10**, 1586 (2019).

5. Shi, E. *et al.* Extrinsic and Dynamic Edge States of Two-Dimensional Lead Halide Perovskites. *ACS Nano* **13**, 1635–1644 (2019).

6. Leng, K. *et al.* Molecularly thin two-dimensional hybrid perovskites with tunable optoelectronic properties due to reversible surface relaxation. *Nat. Mater.* **17**, 908–914 (2018).

7. Stoumpos, C. C. *et al.* High Members of the 2D Ruddlesden-Popper Halide Perovskites: Synthesis, Optical Properties, and Solar Cells of (CH3(CH2)3NH3)2(CH3NH3)4Pb5I16. *Chem* **2**, 427–440 (2017).
